# Changes in Plasma Fatty Acids, Free Amino Acids, Antioxidant Defense, and Physiological Stress by Oleuropein Supplementation in Pigs Prior to Slaughter

**DOI:** 10.3390/antiox9010056

**Published:** 2020-01-08

**Authors:** Ana I. Rey, Almudena de-Cara, Luis Calvo, Patricia Puig, Teresa Hechavarría

**Affiliations:** 1 Dpto. Producción Animal, Facultad de Veterinaria, Universidad Complutense de Madrid, Avda. Puerta de Hierro s/n., 28040 Madrid, Spain; dena.decm@gmail.com; 2 Incarlopsa, Ctra. N-400 km. 95400, 16400 Tarancón, Cuenca, Spain; luiscalvo@incarlopsa.es; 3 Andres Pintaluba, S.A. Polígono Industrial Agro-Reus Prudenci Bertrana, 5, 43206 Reus, Spain; ppuig@pintaluba.com (P.P.); thechavarria@pintaluba.com (T.H.)

**Keywords:** oleuropein, oxidative status, free fatty acids, plasma amino acids, cortisol, pigs, fasting, slaughter

## Abstract

Olive tree leaves are characterized for having not only a potent antioxidant power but also effects on glucose and lipid metabolism. The impact of the individual oleuropein (OLE), vitamin E + Se (VE), or a combined supplementation of oleuropein, vitamin E, and selenium (VEOLE) was evaluated on pig plasma metabolites under fasting prior to slaughter. VEOLE and OLE had lesser n-3 plasma polyunsaturated fatty acids and greater monounsaturated free fatty acids compared to control. The n-3-fatty acid mobilization was directly correlated with greater cystine and inversely with oxidized glutathione/reduced glutathione (GSSH/GSH) levels. This faster use of n-3 fatty acids might act as an indicator of glutathione synthesis mediated by an increase of cystine in plasma. Different correlations and linear adjustments were observed between plasma antioxidant power and free cystine, free glycine, free glutamine, monounsaturated free fatty acids, and total n-3. The best response to stress was found in VEOLE. Cortisol reached the greatest positive correlation with plasma total n-3 fatty acids, which suggests a faster uptake of n-3 for biological functions such as stress control or energy supply in the brain. From a practical point of view, an enhanced oxidative status as well as control of physiological stress prior to slaughter by the combined antioxidants supplementation might have positive effects on pork quality.

## 1. Introduction

Stressful situations for the animal, such as husbandry practices, environmental changes, transport, lairage, and fasting prior to slaughter, result in an imbalance between antioxidant defense and free radical production in the organism [1]. This oxidative stress can induce loss of health status with fails in the immune system, lower productivity and welfare [2] that results in lower quality of the products. 

Dietary supplementation with antioxidants has shown to be an effective strategy to control oxidative stress in vitro [3], in vivo, and meat quality post-mortem [4,5,6]. Much attention has been focused in recent years on the positive effects of natural antioxidants such as those derived from the olive tree (*Olea europaea* L.), whose cultivation has a relevant economic importance in the Mediterranean countries. More than 15 different phenolic antioxidant compounds have been found in olive leaves, and among them one of the most abundant is oleuropein [4]. Oleuropein has shown to exhibit not only antioxidant [7] but also hypolipidemic and hypoglucemic [8] effects. The mode of action of oleuropein is mainly explained by its capacity to catch free radicals and chelating metal ions [9]; however, the molecular mechanism which acts on glucose metabolism is still not clear. It has been suggested that it activates glucose consumption in a dose-dependent manner in adipocytes and in myotubes [10], and may reduce lipid levels by suppressing lipid synthesis and promoting fatty acid oxidation via the regulation of adenosine monophosphate-activated protein kinase (AMPK) [11]. Recently, it has been reported that olive extracts may modulate cannabinoid receptor (CB1) gene expression, which regulates energy homeostasis in the organism [12]. Due to its effects shown on glucose uptake and lipid metabolism, its use by dietary interventions might affect a specific fatty acid mobilization and glycogen stores as well as amino acid utilization [13]; however, there is no information on the specific mobilization of the fatty acid or free amino acid profile prior to slaughter in fasting conditions. This is a relevant aspect since it has been reported that some amino acids not only have influence on antioxidant defense ability [14], but they also modulate certain neuropeptides in the central nervous system being responsible for wellness [15,16]. On the other hand, a different fatty acid utilization may also modify the final profile of meat products, and it has been reported that plasma polyunsaturated fatty acids (PUFA) levels may affect hypothalamic–pituitary–adrenal axis and glucocorticoid concentrations (e.g., cortisol), which is a good indicator of the physiological stress response [17].

Vitamin E is a well-known potent antioxidant against oxidative stress scavenging reactive oxygen species and then maintaining the redox balance in the organism [18]. Vitamin E has shown a synergistic effect with selenium (Se), this combination being more effective to reduce lipid oxidation than the unique administration of one of the antioxidants [5,19]. Selenium plays an important role in antioxidant defenses as a component of Se-dependent glutathione peroxidase which protects cells and contributes to the antioxidant balance [1]. It has been reported in mice that it regulates adipogenesis and lipolysis so its supplementation may prevent fat accumulation in non-obese individuals [20].

Some previous research has compared the effects of dietary vitamin E with olive extracts [21] or specifically oleuropein [6], but these studies were focused on lipid oxidation without any information on the stress response and fatty acid and protein metabolism prior to slaughter. Moreover, there is a lack of information on the possible combined effects of both antioxidants and selenium in fasting conditions prior to slaughter and how these might affect metabolites mobilization. Therefore, the aim of this study was to investigate the effect of dietary oleuropein (96 mg/kg feed) in comparison with 100 mg α-tocopheryl acetate/kg feed + 0.26 mg selenium/kg feed and the oleuropein double-dose combination with vitamin E + Se on oxidative status, physiological stress response (cortisol level), blood fatty acid mobilization, and free amino acid profile in pigs at the time of slaughter in order to gain a better understanding of the mode of action, and secondly, if plasma nutritional profile after dietary oleuropein could predict the oxidative status and well-being prior to slaughter.

## 2. Materials and Methods

All experimental procedures performed in this study complied with Spanish policy for Animal Protection (RD53/2013), which is in accordance with the European Union Directive 2010/63/UE on the protection of animals used for research and with the research committee of the Veterinary Faculty of Complutense University of Madrid.

### 2.1. Animals, Experimental Diets, and Sample Collection

Forty-eight (24 barrows and 24 females) Large White × Landrace pigs were randomly selected at an average live weight of 60.2 ± 1.5 kg, assigned into four groups of 12 pigs each (6 males and 6 females per treatment), and located in a farm with controlled environment (Copiso, Centenera del Campo, Soria, Spain). During the experimental period (last 35 days of fattening), pigs were fed ad libitum on a commercial-based diet (formulated according to National Research Council requirements for pigs) that was identical for all the experimental groups except for the added antioxidant compound (Table A1). Dietary treatments consisted of (1) control (C) (without additional supplementation), (2) 100 mg vitamin E/kg feed + 0.26 mg Se/kg feed (VE), (3) 96 mg oleuropein extract/kg feed (OLE), (4) 100 mg vitamin E/kg feed + 0.26 mg Se/kg feed + 192 mg oleuropein extract/kg feed (VEOLE) (Table A1). Vitamin E (dl-α-tocopheryl acetate 50%), Se (sodium selenite 44%), and oleuropein extract (20%), were provided by Pintaluba S.A. (Reus, Spain) and feeds were formulated and fabricated in Copiso (Soria, Spain).

The pigs were slaughtered after 17 h of fasting and CO_2_ stunning at a commercial abattoir (Incarlopsa, Tarancón, Cuenca, Spain). At the slaughter time, blood samples (*n* = 10 per treatment, *n* = 5 males and *n* = 5 females per treatment) were taken from the jugular vein and collected in tubes with or without coated anticoagulant (Biospin, S.A. Madrid, Spain). All blood samples were immediately placed on ice after collection. The serum and plasma were then separated by centrifugation at 600× *g* for 10 min at 4 °C and the supernatant was immediately frozen on liquid N_2_ and kept in a freezer at −80 °C until analysis. Analyses were carried out within the next 1 month after slaughter.

### 2.2. Laboratory Analysis

#### 2.2.1. Biochemical Parameters

Glucose quantification was measured using a Glucose oxidase/peroxidase (GOD-PAD) kit (Materlab, Madrid, Spain) following manufacturer instructions. Triglycerides were measured using enzymatic kits (Materlab, Madrid, Spain) following manufacturer instructions. Cortisol was determined using chemiluminescent immunoassay technology using manufacturer instructions (Shenzhen Mindray Bio-Medical Electronics Co., Ltd., Shenzhen, China)

#### 2.2.2. Antioxidant Status

The ferric reducing antioxidant power (FRAP) was measured using the procedure described by Benzie and Strain [22] in serum samples. The FRAP reagent consisted of ferric chloride (20 mM) and TPTZ solution (2,4,6-tripyridyl-s-triazine in 40 mM HCl) in acetate buffer (300 mM). Sample extract (100 μL) was mixed with 3 mL of the working FRAP solution (3 mL). The absorbances at 593 nm were measured 0 and 4 min after the mixture. Results were calculated as μM.

The levels of glutathione (reduced: GSH, and oxidized forms: GSSH) were measured using a glutathione colorimetric detection kit (Arbor Assays, Ann Arbor, MI, USA) according to manufacturer’s instructions after treatment with 2-vinylpyridine. Free glutathione (GSH) concentrations were obtained by subtracting the oxidized glutathione (GSSH) levels obtained from the 2-vinylpyridine-treated standard from nontreated standards and samples (total GSH). The concentrations were calculated as µM of glutathione.

The α-tocopherol concentration in the pigs’ serum was quantified as described by Rey et al. [23]. Tocopherol was extracted by a direct procedure after mixing with hexane from serum samples (in duplicate) in the presence of a dibasic sodium phosphate buffer (0.054 M) and absolute ethanol. Detection of α-tocopherol was performed by using an HPLC (HP 1100, Agilent Technologies, Waldbronn, Germany), equipped with a diode array detector and a reverse column (RP-18, Agilent Technologies). A standard curve using the pure compound (Sigma, Alcobendas, Madrid) was built to quantify the µg of α-tocopherol per mL of plasma.

Serum levels of the thiobarbituric acid reactive substances (TBARS) were measured spectrophotometrically as the reaction products of malondialdehyde (MDA) with thiobarbituric acid [24]. MDA concentrations were calculated using 1.56 × 10^5^ M^−1^ × cm^−1^ as the molar absorption coefficient. Results were expressed as mmoles MDA/L serum.

#### 2.2.3. Plasma Free Amino Acid Profile

Plasma free amino acid profile was analyzed following the procedure of Bruce et al. [25]. Briefly, a mixture of acetonitrile:methanol:acetone was added to 100 µl of plasma and the supernatant was mixed with methanol to discard the superior phase. After evaporation of the solvents with N_2_ stream, samples were redisolved in water (MilliQ). Samples (1 µL) were derivatized using o-ortho-ftalaldelhide (o-phthalaldehyde) reagent in 0.4 mM borate buffer and 40 µl of Na_2_HPO_4_ as diluent agent in an HPLC (Agilent 1100, 1046A) equipped with a fluorescent detector, a phase reverse column Porshell HPH-C18 (4.6 × 100 mm, 2.7 µm, Agilent Technologies, Germany), and a pre-column HPH-C18 (Infinitylab Porshell 120, 3.0 mm, UHPLC, Agilent Technologies, Germany). Derivatized samples (20 µL) were injected in two different mobile phases following a gradient of 1.5 mL/min. The mobile phase A was a dilution of Na_2_HPO_4_, 10 mM Na_2_B_4_O_7_ (pH 8.2), and 0.5 mM NaN_3,_ and the mobile phase B was a mixture of acetonitrile:methanol:water (45:45:10). The fluorescent detector was fixed at 340 nm for excitation and 450 nm for emission. Amino acids were identified by comparison of their retention times with those of a standard mixture of 17 l-amino acids: Alanine, arginine, aspartic acid, cystine, glutamic acid, glycine, histidine, isoleucine, leucine, lysine, methionine, phenylalanine, proline, serine, threonine, tyrosine, and valine (1 nm/µL in HCl 0.1 M, Agilent Technologies) and a dilution of asparagine and glutamine (0.01 M HCl) and tryptophan (0.1 M HCl, Agilent Technologies).

#### 2.2.4. Plasma Fatty Acid Profile

Plasma lipids were extracted in lyophilized samples as described by Segura and López-Bote [26]. Samples were mixed (MM400, Retsch technology, Haan, Germany) in the presence of a mixture of dichloromethane-methanol (8:2) and centrifugated (8 min at 10,000 rpm) to obtain the upper phase containing lipids. Fatty acids in the total lipid extracts were identified and quantified by gas chromatography (HP6890, Hewlett Packard, Avondale, PA, USA) after methylation [27]. Neutral lipids (NL) and free fatty acids (FFA) were separated from the total lipid extracts using aminopropyl minicolumns (Varian, Harbor City, CA, USA), which had been previously activated with hexane (7.5 mL) [28]. After fat addition (20 mg dissolved in hexane:chloroform:methanol, 95:3:2), NL were extracted with chloroform (5 mL) and FFA with diethylether:acetic acid (98:2) (5 mL). Esterification to obtain fatty acids methyl esters was carried out at 80 °C for 1 h in the presence of 3 mL of methanol:toluene:H_2_SO_4_ (88:10:2) [27]. The fatty acid methyl esters were separated by gas chromatograph (HP 6890, Hewlett Packard, Avondale, PA, USA) equipped with flame ionization detector and a capillary column (HP-Innowax Polyethylene Glycol, 30 m length × 0.316 mm × 0.25 µm). Nitrogen was used as a carrier gas at a flow rate of 2.3 mL/min. After injection (1 µL) at 170 °C and split 1/50, the oven temperature was raised to 210 °C at a rate 3.5 °C/min, then to 250 °C at a rate of 7 °C/min and held constant for 1 min. The detector was held at constant temperature of 250 °C. For peak identification, a standard mix of fatty acid methylesters (Sigma-Aldrich, Alcobendas, Spain) was used.

The relative mobilization index was calculated as the ratio between the percentage in free fatty acids and neutral lipids (triacylglycerols) [29].

#### 2.2.5. Statistical Analysis

The experimental unit for analysis of all data was the pig. Data were analyzed following a completely randomized design using the general linear model (GLM) procedure contained in Statistical Analysis System (SAS, version 9; SAS Inst. Inc., Cary, NC, USA). Dietary treatment was considered the fix effect. Homogeneity of variance was checked prior to analysis. Data were presented as the mean of each group and the mean standard error (SEM) together with significance levels (*P* value) of the main effect. Tukey’s test was used to separate treatment means. Differences between means were considered statistically significant at *P* < 0.05. Pearson correlation analyses (Statgraphics-18 program) were carried out among plasma fatty acids and amino acids and between antioxidant status indicators (FRAP, GSSH/GSH) or physiological stress (cortisol) and main fatty acids: saturated (SAT), monounsaturated (MUFA), and polyunsaturated (PUFA) and amino acids. Two significant P values levels for correlation analyses were considered (*P* < 0.05 and *P* < 0.01). Linear adjustment between variables was carried out by means of the Statgraphics-18 program.

## 3. Results

### 3.1. Biochemical Parameters of Blood Samples and Oxidative Status

Serum glucose was clearly diminished by dietary oleuropein supplementation (OLE and VEOLE) (Table 1) after fasting prior to slaughter (*P* = 0.0175). This effect was more marked when oleuropein was administered independently than when it was combined with vitamin E and selenium. Serum triglycerides tended to be lower by oleuropein supplementation (*P* = 0.09) but differences were not statistically significant. Cortisol level at the time of slaughter was unaffected by the single supplementation of the antioxidants (VE, OLE) when compared to the control group. However, the combination of antioxidants (VEOLE) resulted in a 50% decrease in its values (*P* = 0.0001).

The oxidative status of blood samples is presented in Table 1. Serum from pigs supplemented with antioxidants or its combination tended to have lesser TBARS values when compared to the control (*P* = 0.056). Moreover, FRAP was greater (*P* = 0.0014) in those groups supplemented with oleuropein when compared to the control and had intermediate values in the VE group. Alpha-tocopherol concentration of the serum samples was not affected by dietary oleuropein.

The different forms of glutathione were also measured in plasma (Table 1). Dietary oleuropein reduced GSSH production (*P* = 0.0001) when compared to the other groups, which resulted in a lower GSSH/GSH ratio (*P* = 0.0001). The group with the greatest reduced form of glutathione was VEOLE (*P* = 0.0001), and this also had the lowest GSSH form and consequently the lowest GSSH/GSH ratio. VE group had intermediate GSSH values and GSSH/GSH ratio when compared to the others.

### 3.2. Free Amino Acids in Plasma

The profile of free amino acids of plasma samples is presented in Table 2. Cystine and lysine were affected by antioxidant supplementation (*P* < 0.0001). Hence, VE, OLE, and VEOLE groups had greater free cystine and lesser free lysine concentrations in plasma when compared to control.

### 3.3. Total Fatty Acid Profile, Free Fatty Acids, and Fatty Acid Mobilization Index in Plasma

The total fatty acid profile of the plasma samples is presented in Table 3. The group VEOLE had greater proportions of C18:0 (*P* = 0.018) and C20:1n-9 (*P* = 0.001), whereas it observed a reduction in C17:1 (*P* = 0.027), C16:1n-9 (*P* = 0.0001), C20:5n-3 (*P* = 0.004), and C22:6n-3 (*P* = 0.039), when compared to control group. Also, C22:5n-3 tended to be lesser (*P* = 0.064) in VEOLE when compared to control. As a result, the proportion of SAT was the greatest in the group VEOLE when compared to control and VE, while the single supplementation of oleuropein (OLE) reached intermediate values (*P* = 0.021). Moreover, it observed a decrease in the proportion of total n-3 PUFA (*P* = 0.0004) that reached the lowest proportion in VEOLE followed by OLE, VE, and control groups. The n-6 fatty acids also tended to be lesser (*P* = 0.064) in groups supplemented with oleuropein. Consequently, the group with the greatest n-6:n-3 ratio was VEOLE (*P* = 0.015) when compared with the other groups that did not differ between them.

In order to study fatty acid mobilization, the free fatty acid fraction of the lipid profile of plasma samples was quantified (Table 4). The oleic acid was the fatty acid that was found at greater proportion (*P* = 0.009) in those groups supplemented with oleuropein in diet (OLE and VEOLE), whereas OLE and VEOLE showed lesser C17:0 when compared to control group (*P* = 0.009). These modification in those specific fatty acids produced an increase in the total MUFA proportion of the groups OLE and VEOLE when compared to control (*P* = 0.039), whereas VE group showed intermediate proportions. Moreover, the saturated fatty acid proportion tended to be lesser in OLE and VEOLE (*P* = 0.15). No clear effect of the oleuropein supplementation on other fatty acids was detected. A tendency to intermediate proportions of C14:0 (*P* = 0.09), C16:1n-7 (*P* = 0.061), C18:3n-6 (*P* = 0.070), and C22:1n-9 (*P* = 0.056) in groups OLE and VEOLE was observed when compared to the control. No clear differences were found between VE and oleuropein supplemented groups.

**Figure 1:**
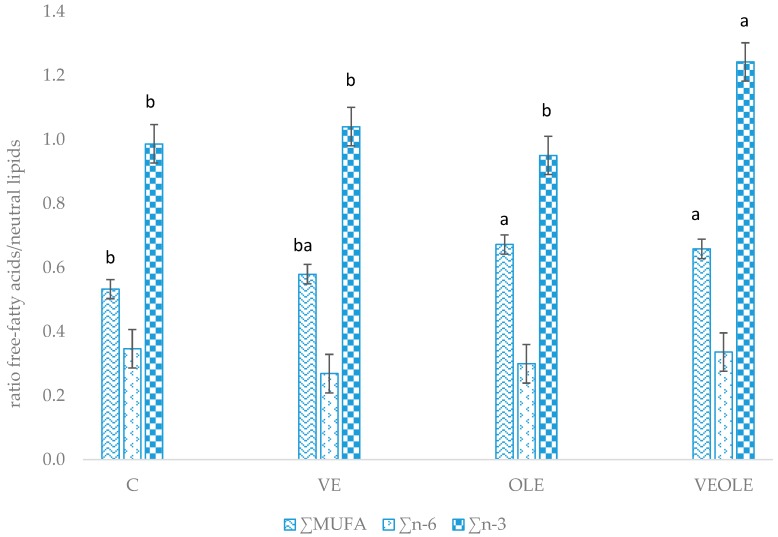
Main fatty acids relative mobilization index (plasma free fatty acids/plasma neutral fatty acids). Different letters (a, b) indicated *P* < 0.05

The relative mobilization index (calculated as the ratio between free fatty acid:neutral lipids of plasma) (Figure 1) showed greater ratios for MUFA in OLE and VEOLE groups when compared to control. VE group had intermediate MUFA mobilization that did not differ from the other groups. Moreover, VEOLE had the greatest n-3 fatty acid mobilization index when compared to the others.

### 3.4. Correlations between Metabolites

**Figure 2:**
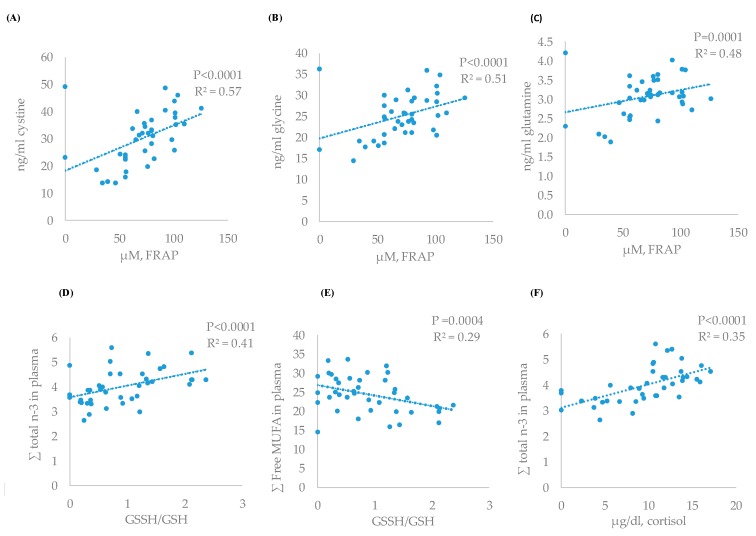
Relationship between antioxidant status and amino acids or fatty acid profile (linear): FRAP and cystine (**A**), FRAP and glycine (**B**), FRAP and glutamine (**C**), GSSH/GSH and n-3 (**D**), GSSH/GSH and free MUFA (**E**), and cortisol and n-3 fatty acids (**F**) of the experimental results.

The greatest correlation between cortisol and blood metabolites was found with the total of n-3 fatty acids (r = 0.49) (Table 5) that showed a linear adjustment (Figure 2F, *P* < 0.0001, R^2^ = 0.35), followed by cystine (r = −0.36, *P* = 0.037). Cortisol also showed greater correlation with GSSH/GSH ratio (r = 0.60) than FRAP (r = −0.36).

Also, a direct correlation between the antioxidant power (FRAP) and different amino acids was found (Table 5). The greatest direct correlations were found for cystine (r = 0.75), glycine (r = 0.60), and glutamine (r = 0.54). The response of these free amino acids and FRAP concentrations showed significant and linear adjustments (*P* < 0.05, R^2^ = 0.57, R^2^ = 0.51, and R^2^ = 0.48 for glycine, cystine, and glutamine, respectively) (Figure 2A–C). Similar inverse relations between these amino acids and GSSH/GSH ratio were detected but magnitude of correlation and adjustment were lesser than for total antioxidant power.

Finally, a direct correlation and linear response were found between plasma total n-3 fatty acids and GSSH/GSH ratio (r = 0.50, *P* < 0.0001, R^2^ = 0.35) (Table 5, Figure 2C) and inverse between free MUFA fatty acids and GSSH/GSH (r = −0.56, *P* < 0.0004, R^2^ = 0.29) (Table 5, Figure 2D). Moreover, the decrease in n-3 fatty acids was positively correlated with greater cystine values and a linear adjustment between these two variables (r = −0.49, *P* = 0.0014, R^2^ = 0.23) (Table 5) was found. The increase in saturated, as a consequence of PUFA decrease, was also inversely related with lower tyrosine concentrations (r = −0.39, *P* = 0.014) and the increase in free MUFA fatty acids with positive changes (*P* < 0.05) in cystine (r = 0.33), valine (r = 0.44), leucine (r = 0.36), and isoleucine (r = 0.47).

## 4. Discussion

Given the evidence of the multiple positive effects of oleuropein extract in human and rat studies, we decided to test the effect of oleuropein at different doses and in combination with other well-known antioxidant substances such as vitamin E in growing–finishing pigs. The aim was to study the effects on the metabolic and oxidative status and on the physiologic stress response during fasting prior to slaughter to find the most suitable dose and combination.

Dietary oleuropein supplementation for pigs showed clear effects on the glucose metabolism and on the oxidative status. The antioxidant dose did not enhance this effect. In a previous study [30], monomeric and polymeric substances from olive mill waste in induced diabetic rats were found to decrease the glucose levels in plasma by 55% compared to untreated diabetics. Similar results were reported later with the specific use of oleuropein extracts [8]. The mechanism by which this is explained is not clear enough. According to Hadrich et al. [8] oleuropein induces glucose uptake by the activation of AMP-activated kinase (AMPK), which leads to glucose transporter translocation from the cytosol to the plasma membrane, and consequently, an increase in glucose absorption. Also, it has been indicated that oleuropein was able to stimulate glucose consumption in a dose-dependent manner [8]. However, this dose–effect relationship in diminishing blood glucose levels was not observed in our study when the dietary oleuropein increased to 192 mg/kg in combination with vitamin E and Se. This moderated effect in decreasing the blood glucose of this group (VEOLE) could be explained by its combination with the other antioxidants. Abdel-Raheem et al. [31] found that supplementation of Se and vitamin E increased blood glucose in ewes. Metabolomic analyses of the liver tissue in rats with vitamin E deficiency had lower glucose content than those with a sufficient amount [32]. The effect of selenium on the glucose and lipid metabolism is inconsistent in the literature. According to Luo et al., [33] there is a risk of impaired glucose regulation as the levels of blood selenium increase, whereas other authors have not found negative effects [34]. The results of the present study seem to indicate that higher oleuropein supplementation may counteract the effect of supranutritional Se doses and, finally, result in similar or slightly moderate blood parameters when compared to the unique administration of the oleuropein extract. This effect was also observed in blood triglycerides. Single administration of dietary oleuropein (OLE) tended to decrease serum triglycerides (TG) as observed in other trials in rats [8] and rabbits [34]. However, no differences were found in this parameter in birds [6] when using doses up to 200 mg/kg of oleuropein for 35 days. Differences may be due to the bioavailability of the oleuropein form or the dose–time combination, since Andreadou et al. [35] found differences in TG after a 6-week trial but not after 3 weeks of administration of a higher dose. In the present study, the effect of higher doses of oleuropein supplementation resulted in similar TG concentrations when compared to the use of 100 mg/kg of vitamin E + 0.26 mg/kg of Se. Abdel-Raheem et al. [31] also found TG decreased two weeks postpartum after 450 mg vitamin E and 5 mg Se supplementation twice weekly, similar to Saleh and Ebeid [36] in broilers supplemented with 0.5 mg/kg of Se.

In the present research, the oxidative status of those antioxidant-supplemented animals was more favorable than those of the control group. There were no differences in MDA concentration between dietary oleuropein-supplemented pigs and those given vitamin E and Se; however, the antioxidant capacity of plasma measured as FRAP was greater in those animals that received the oleuropein extract, which indicated its metal-chelating power. The reducing ability of the phenols is due to the number of hydroxyl groups in the aromatic ring. The effectiveness of dietary vitamin E for controlling MDA and FRAP values has already been reported [22,37]; however, it has been found that the contribution of vitamin E to total FRAP was only 5% [22], which could, in part, explain why VEOLE registered no statistically different antioxidant capacity of plasma compared to OLE, even though the ferric reducing abilities of oleuropein were significantly greater than those of ascorbic acid and trolox [38].

It is also interesting to observe a dose-dependent effect on the oxidized forms of glutathione that resulted in a more favorable ratio in those pigs supplemented with oleuropein, vitamin E, and Se (VEOLE), which is important to maintain an adequate redox balance in the organism. These results would be explained by the contribution of the different antioxidants to the glutathione route. Other authors [7,30] also found greater activity of antioxidant enzymes after the administration of olive extracts. The ability of Se as a constituent of the glutathione peroxidase enzyme to increase its activity has also been reported, as well as the effect of vitamin E on the glutathione redox system [37].

Another important nutrient for maintaining the antioxidant defense in the organism that could be affected by the dietary antioxidant supplementation was vitamin E, whose level was also relevant. However, dietary oleuropein did not modify the concentration of serum vitamin E as observed by other authors in literature [39].

The free amino acid profile of plasma was studied to understand the contribution of certain amino acids to the oxidative status. The structure of some antioxidant enzymes such as glutathione, which is one of the most abundant peptides in animal cells, is formed by glycine, cysteine, and glutamine [40]. However, there is no previous information on the effect of oleuropein administration on the plasma amino acid profile. The greater availability of free cystine in those groups supplemented with antioxidants and its linear and negative relationship (r = –0.55, *P* = 0.0005, R^2^ = 0.30) with the oxidized form of glutathione (GSSH) would confirm the gradual and dose-dependent effect of the different dietary antioxidants used in the present study on the cellular antioxidant glutathione system. A similar direct relationship of cystine, glycine, glutamine, and FRAP was also observed. These results would indicate the important contribution of these plasma amino acids to the antioxidant status of the animal [41]. Moreover, the lower concentration of plasma lysine in those groups that received antioxidants is in line with the reported prooxidant effect of this amino acid and its effects on reducing GSH cytoplasmatic and mitochondrial GSH pools [42]. These lower levels of lysine in groups supplemented with antioxidants could be explained in part by the tendency to have greater but not significant plasma arginine concentrations in these groups, since it has been reported that arginine and glucose compete with lysine for brain uptake [43]. In this sense, arginine has been reported to have protective effects on oxidative stress [14].

A detailed study of the fatty acid profile of plasma was carried out to obtain a better understanding of the dietary antioxidants in the lipid metabolism. Plasma total lipids showed that dietary oleuropein extract reduced the proportion of n-3 and, to a lesser extent, n-6-PUFA. Vitamin E + Se also reduced these plasma fatty acids when compared to the control. Plasma did not reflect the fatty acid composition of diets, which would indicate effects of the administered antioxidants on the lipid metabolism. There is a lack of information on how oleuropein or other antioxidants might affect the proportion of circulating fatty acids. This is of interest because a high proportion comes from adipose deposits and this would indicate lipid mobilization to meet the needs of cellular nutrition. In the case of our study on fasting conditions and stressful situations before slaughtering, those groups that showed the lowest PUFA and n-3 fatty acids had a faster glucose consumption and consequently faster lipolysis initiation and triglyceride hydrolysis [29]. González-Santiago et al. [44] found that the administration in rabbits of hydroxytyrosol present in olive oil reduced the proportion of total PUFA and tended to increase MUFA one month after the administration of an atherogenic diet, and this was attributed to a possible increase in the activity of stearoyl-Co-A-desaturase. In order to understand the fatty acid mobilization, we also quantified the fatty acid proportion of FFA in plasma lipids. Blood FFA are hydrolyzed using triglycerides from different locations, mainly adipose tissue. However, in the FFA fraction we only detected greater MUFA (C18:1n-9) in those groups supplemented with oleuropein. These results might indicate that PUFA free fatty acids were rapidly used for energy supply or other functions. A description of the preferred use of PUFA has been given, mainly n-3 from the tissues [29]. Also, Bochicchio et al. [45] found a greater MUFA content but no effects on PUFA in the fatty acid profile of subcutaneous fat in 60 h-fasted pigs. This result could be explained, as in the present study, by a faster utilization of PUFA. This faster use of n-3 fatty acids and consequently greater free MUFA and free SAT might act as indicator of glutathione synthesis mediated by an increase in cystine in plasma, since a high correlation and linear response were found between these fatty acids and cystine as well as GSSH/GSH. Hermier et al. [46] reported the regulating effect of n-3 fatty acids on the amino acid metabolism, with increases in the synthesis of glutathione after the addition of vegetable oils rich in n-3 fatty acids. In the present research, a linear inverse relationship was found between n-3 and cystine and between GSSH/GSH and free MUFA, whereas the relationship was direct between free MUFA and cystine and between GSSH/GSH and n-3 fatty acids that would confirm the importance of the fatty acid and amino acids induced by oleuropein supplementation doses to explain the regulation of its antioxidant function. These results have an important practical impact since an increase in the antioxidant status of the animal or enhanced presence of MUFA free fatty acids prior to slaughter might improve the lipid stability of meat.

Another interesting result of the present study is the correlation observed for the physiological stress response of the animal and plasma n-3 fatty acids. The well-being of the pigs at the time of slaughter was checked by measuring the cortisol level. It is important to note that the combination of dietary oleuropein and the other antioxidants significantly reduced cortisol levels as opposed to the unique administration of these antioxidants at lower doses. As far as we know, there is no information on the possible effects of oleuropein on the cortisol level under fasting and stressful conditions in pigs. It has been reported that oleuropein reduces anxiety responses in rats suffering from post-traumatic stress disorder [47]. Additionally, vitamin E (50 mg/kg) and Se (0.3 mg/kg) reduced cortisol in sheep under heat stress [48]. However, in pigs, following transport stress, the supplementation of 200 mg/kg of vitamin E was not enough to reduce cortisol, with supplementation with oregano essential oil having a greater effect. According to the results of the present study, the administration of higher doses of oleuropein together with the other antioxidants would be an interesting strategy to reduce stress before slaughter. The mode of action is still unclear. It has been suggested that oleuropein may alter catecholamine synthesis in the brain [47]. On the other hand, it has been reported that hydroxytyrosol, another antioxidant substance extracted from olive leaves, may affect the endocannabinoid system by inhibition of its receptors (CB1) [12]. The endocananabonoid system is not only involved in energy regulation in the organism as a metabolic signaling system, but also in the stress response [17]. Hence, it has been suggested that a higher PUFA n-6:n-3 ratio in neurons may produce over-activation of the endocannabinoid system in the limbic areas that control emotions [17]. Other studies show that n-3 fatty acids inhibit adrenal activation related to stress probably through effects on the central nervous system [49]. Plasma n-3 fatty acids are transported through the blood–brain barrier and represent the most important source for nerve cells [50]. Thus, the lower plasma n-3 fatty acids of the group supplemented with the higher dose of oleuropein would indicate a faster uptake for energy supply or biological functions such as those described in the brain for stress control in stressful situations. These results did not coincide with those observed by Nemeth et al. [51], in which saliva cortisol concentrations were diminished by higher plasma n-3 PUFA. However, these authors [51] reported that the nutritional status was involved in the regulation of the hypothalamic–pituitary–adrenal (cortisol production) in the short term, whereas a greater n-3 mobilization due to stressful situations, as observed in the present study under fasting for several hours, could also modulate cortisol production in the long term. In addition, in the present study, changes in n-3 fatty acids induced a greater proportion of total and free saturated fatty acids and increases in these fatty acid fractions were correlated with less tyrosine (amino acid that has been a precursor of catecholamine synthesis). A decrease in blood tyrosine concentration raises the brain uptake of other large neutral amino acids like tryptophan through competition for the common transporter and consequently cognition or cortisol secretion may be affected [16]. However, the unique compound that had a direct correlation with cortisol was cystine. In addition, a high correlation between cortisol and the glutathione ratio was detected and would once again indicate the interaction between plasma n-3 and glutathione formation confirming the possibility that changes in the nutritional profile induced by substances such as oleuropein could control physiological stress.

## 5. Conclusions

To conclude, the supplementation of oleuropein at 192 mg/kg in combination with vitamin E and selenium for 35 days reduced serum glucose and counteracted the possible opposite effects of Se on the glucose metabolism. A higher oleuropein concentration with the other antioxidants was also more effective to enhance the oxidative status of the animal mainly by an increase in the glutathione route. This combination also modified the nutritional profile of plasma that resulted in faster n-3 fatty acid mobilization. An oleuropein dose–response (linear adjustment) of the proportion of plasma n-3, cystine concentration, and oxidized glutathione was observed and indicates the possible regulation effect of fatty acid proportions on glutathione synthesis mainly mediated by cystine. The highest dose of oleuropein in combination with the other antioxidants was very effective in reducing cortisol levels under fasting conditions prior to slaughter. The faster utilization of the n-3 fatty acids in this group could modulate cortisol production. It would be possible to predict the antioxidant status and the well-being of the animal from the plasma nutritional profile (mainly levels of cystine, glycine, or glutamine and total n-3 and free MUFA for the antioxidant status; and free-n-3 for physiological stress). However, to obtain validated equations, further data would be needed for different dietary supplements. From a practical point of view, an enhanced oxidative status and presence of some nutrients (MUFA fatty acids, cystine) as well as control of physiological stress prior to slaughter by oleuropein + vitamin E + selenium supplementation might have very positive effects on meat stability and quality of products.

From the practical point of view, an increase in the oxidative status and the physiological control of stress along with the presence of antioxidant nutrients (cystine, MUFA fatty acids) as a consequence of oleuropein supplementation could have very positive effects on the meat stability.

## Figures and Tables

**Table 1:** Biochemical parameters and oxidative status of blood samples at slaughter from pigs fed the experimental diets (C, control; VE, vitamin E + Se; OLE, oleuropein extract; VEOLE, vitamin E + Se + oleuropein extract).

Dietary Treatment	C	VE	OLE	VEOLE	SEM ^1^	P ^2^
Biochemical parameters of serum at slaughter						
Cortisol. µg/dL	12.73 ^a^	12.76 ^a^	11.92 ^a^	6.36 ^b^	0.84	0.0001
Glucose. mmoles/dL	178.90 ^ba^	183.40 ^a^	143.22 ^c^	157.10 ^bc^	9.37	0.0175
Triglycerides. mg/100mL	90.80	82.10	74.25	84.43	4.51	0.0950
Oxidative status of blood samples						
Alpha-tocopherol. µg/g	1.29 ^b^	1.45 ^a^	1.28 ^b^	1.49 ^a^	0.03	0.0001
FRAP. µM	55.80 ^b^	73.97 ^ba^	96.57 ^a^	99.84 ^a^	5.90	0.0014
TBARS. mmoles/L	0.02	0.01	0.01	0.01	0.00	0.0567
GSSH. µM	6.54 ^a^	5.78 ^a^	3.82 ^bc^	2.58 ^c^	0.39	0.0001
Free GSH. µM	4.56 ^b^	4.75 ^b^	5.15 ^b^	7.67 ^a^	0.40	0.0001
GSSH/GSH	1.53 ^a^	1.41 ^a^	0.81 ^b^	0.35 ^c^	0.13	0.0001

^1^ SEM: Mean standard error, *n* = 10; ^2^ P: Letters with different superscript were statistically significant.

**Table 2:** Amino acid profile of plasma samples at slaughter from pigs fed the experimental diets (C, control; VE, vitamin E + Se; OLE, oleuropein extract; VEOLE, vitamin E + Se + oleuropein extract).

Dietary Treatment	C	VE	OLE	VEOLE	SEM ^1^	P ^2^
Aminoacid Profile of Plasma Samples (ng/mL)						
Aspartic acid	5.77	5.72	5.24	5.15	0.474	0.7101
Glutamic acid	82.58	87.66	81.21	90.34	6.767	0.7539
Asparagine	0.3	0.28	0.28	0.29	0.013	0.6341
Serine	14.14	14.11	13.77	15.61	0.822	0.4048
Glutamine	2.87	3.08	3.19	3.16	0.168	0.5378
Histidine	3.37	3.39	3.99	3.56	0.316	0.4807
Glycine	24.37	25.06	24.5	26.52	1.701	0.8015
Threonine	16.42	15.63	16.23	15.58	0.860	0.8671
Arginine	1.25	2.38	2.41	1.66	0.373	0.0982
Alanine	41.87	37.92	41	46.82	2.716	0.1563
Tyrosine	10.09	9.36	9.21	9.46	0.576	0.7197
Cystine	18.8 ^b^	32.86 ^a^	32.52 ^a^	36.5 ^a^	2.179	0.0001
Valine	11.4	11.33	12.06	12.3	0.484	0.4081
Methionine	4.05	3.99	3.83	4.16	0.253	0.8331
Tryptophan	0.79	0.73	0.7	0.73	0.035	0.3597
Phenylalanine	11	10.19	10.2	10.52	0.345	0.3207
Isoleucine	12.89	12.12	12.65	13.46	0.512	0.3317
Leucine	17.71	16.73	17.86	18.1	0.645	0.4614
Lysine	23.66 ^a^	16.49 ^b^	16.87 ^b^	17.2 ^b^	0.911	0.0001
Essentials ^3^	102.52	92.97	96.56	97.27	3.349	0.264
Non essentials ^4^	200.8	216.04	210.93	233.84	12.700	0.3288

^1^ SEM: Mean standard error, *n* = 10; ^2^ P: Letters with different superscript were statistically significant; ^3^ essentials: Sum of histidine, threonine, valine, methionine, tryptophan, phenylalanine, isoleucine, leucine, and lysine; ^4^ non-essentials: Sum of aspartic and glutamin acids, asparagine, serine, glutamine, glycine, alanine, tyrosine, arginine, and cystine.

**Table 3:** Effect of dietary oleuropein (OLE), vitamin E and selenium (VE), or their combination (VEOLE) on the total fatty acid profile (g/100 g total fatty acids) of plasma samples from pigs fed the experimental diets.

Fatty Acids	C	VE	OLE	VEOLE	SEM ^1^	P ^2^
C14:0	0.87	0.83	0.97	0.99	0.074	0.3830
C16:0	20.58	20.43	20.98	21.22	0.324	0.3071
C16:1n7	1.38	1.45	1.45	1.51	0.058	0.4865
C16:1n9	0.66 ^a^	0.64 ^a^	0.38 ^c^	0.51 ^b^	0.033	0.0001
C17:0	0.53	0.50	0.56	0.54	0.044	0.8140
C17:1	0.64	0.62	0.52	0.49	0.040	0.0268
C18:0	17.75 ^b^	18.06 ^ba^	18.83 ^ba^	19.88 ^a^	0.487	0.0181
C18:1n7	1.67	1.75	1.94	1.91	0.081	0.0796
C18:1n9	23.76	23.95	24.68	23.10	0.570	0.2903
C18:2n6	17.01 ^a^	16.80 ^ba^	15.37 ^b^	15.87 ^ba^	0.426	0.0309
C18:3n3	0.68 ^ba^	0.71 ^a^	0.60 ^b^	0.60 ^b^	0.024	0.0041
C18:3n6	0.43 ^a^	0.39 ^ba^	0.30 ^b^	0.37 ^ba^	0.029	0.0324
C20:0	0.24 ^ba^	0.27 ^a^	0.18 ^b^	0.16 ^b^	0.022	0.0050
C20:1n9	0.22 ^b^	0.28 ^ba^	0.24 ^b^	0.37 ^a^	0.025	0.0010
C20:3n6	0.45	0.40	0.34	0.41	0.034	0.2036
C20:4n6	7.87	7.85	7.09	7.92	0.378	0.3675
C20:5n3	0.41 ^a^	0.38 ^ba^	0.25 ^b^	0.24 ^b^	0.039	0.0039
C22:1	0.65	0.71	0.77	0.70	0.077	0.7388
C22:4n6	0.72	0.84	0.76	0.65	0.068	0.2697
C22:5n3	1.94	1.81	1.59	1.52	0.120	0.0648
C22:6n3	1.51 ^a^	1.34 ^ba^	1.38	1.05 ^b^	0.112	0.0389
∑SAT ^3^	39.98 ^b^	40.09 ^b^	41.54 ^ba^	42.79 ^a^	0.696	0.0211
∑MUFA ^4^	28.99	29.41	29.99	28.59	0.595	0.3996
∑PUFA ^5^	31.03 ^a^	30.50 ^ba^	27.70 ^b^	28.62 ^ba^	0.774	0.0134
∑n-6 ^6^	26.49	26.26	23.88	25.22	0.734	0.0642
∑n-3 ^7^	4.55 ^a^	4.23 ^ba^	3.82 ^bc^	3.40 ^c^	0.180	0.0004
∑n-6:∑n-3	5.97 ^b^	6.33 ^ba^	6.34 ^ba^	7.47 ^a^	0.326	0.0149

^1^ SEM: Mean standard error, *n* = 10: ^2^ P: Letters with different superscript were statistically significant; ^3^ SAT: Sum of saturated fatty acids; ^4^ MUFA: Sum of monounsaturated fatty acids; ^5^ PUFA: Sum of polyunsaturated fatty acids; ^6^ ∑n-6: Sum of n-6 polyunsaturated fatty acids; ^7^ ∑n-3: Sum of n-3 polyunsaturated fatty acids.

**Table 4:** Effect of dietary oleuropein (OLE), vitamin E and selenium (VE), or their combination (VEOLE) on the free fatty acid fraction (g/100 g total fatty acids) of plasma samples from pigs fed the experimental diets.

Free Fatty Acids	C	VE	OLE	VEOLE	SEM ^1^	P ^2^
C14:0	1.85	1.44	1.55	1.60	0.116	0.0946
C16:0	28.65	30.34	28.01	28.23	0.802	0.1790
C16:1n7	1.94	1.43	1.54	1.55	0.136	0.0611
C16:1n9	0.74	0.71	0.67	0.63	0.044	0.3532
C17:0	3.12 ^a^	1.69 ^b^	1.69 ^b^	1.58 ^b^	0.350	0.0095
C17:1	0.21	0.16	0.16	0.21	0.020	0.1331
C18:0	32.92	33.71	31.68	30.58	1.248	0.3190
C18:1n7	1.54	1.54	1.47	1.40	0.110	0.7887
C18:1n9	16.55 ^b^	18.60 ^ba^	21.79 ^a^	22.32 ^a^	1.293	0.0093
C18:2n6	8.56	6.67	7.83	7.86	0.658	0.2549
C18:3n3	0.55	0.54	0.62	0.67	0.045	0.1507
C18:3n6	0.27	0.22	0.23	0.26	0.015	0.0701
C20:0	0.28	0.29	0.29	0.33	0.024	0.3803
C20:1n9	0.57	0.49	0.57	0.59	0.028	0.0767
C20:4n6	0.85	0.79	0.84	0.91	0.057	0.4862
C20:5n3	0.71	0.74	0.58	0.68	0.062	0.2857
C22:1n9	0.69 ^a^	0.67 ^a^	0.48 ^b^	0.60 ^ba^	0.057	0.0561
∑SAT ^3^	66.83	67.46	63.22	62.32	1.881	0.1543
∑MUFA ^4^	21.54 ^b^	22.93 ^ba^	26.21 ^a^	26.70 ^a^	1.429	0.0394
∑PUFA ^5^	10.94	8.95	10.10	10.38	0.714	0.2658
∑n-6 ^6^	9.68	7.68	8.90	9.04	0.706	0.2571
∑n-3 ^7^	1.26	1.28	1.20	1.35	0.059	0.3682
∑n-6:∑n-3	7.63	6.26	7.53	6.89	0.599	0.3504

^1^ SEM: mean standard error, *n* = 10; ^2^ P: Letters with different superscript were statistically significant; ^3^ SAT: Sum of saturated fatty acids; ^4^ MUFA: Sum of monounsaturated fatty acids; ^5^ PUFA: Sum of polyunsaturated fatty acids; ^6^ ∑n-6: Sum of n-6 polyunsaturated fatty acids; ^7^ ∑n-3: Sum of n-3 polyunsaturated fatty acids.

**Table 5:** Correlation coefficients between plasma amino acids and fatty acids and between antioxidant status: ferric reducing antioxidant power (FRAP) and oxidized glutathione/reduced glutathione (GSSH/GSH) or physiological stress (cortisol) and different metabolites in plasma.

Free-Aminoacids	SAT	MUFA	n-6	n-3	Free-SAT	Free-MUFA	Free-n-6	Free-n-3	FRAP	GSSG/GSH	Cortisol
Asp	−0.18	0.13	−0.03	0.08	0.24	−0.17	−0.30	0.15	0.18	−0.04	0.26
Glu	0.01	0.19	−0.20	−0.21	0.11	−0.02	−0.26	0.11	0.48 ^a^	−0.20	0.06
Asn	−0.14	0.06	0.23	−0.08	−0.08	0.20	−0.21	0.05	−0.37	−0.27	0.00
Ser	0.09	0.08	−0.02	−0.28	−0.10	0.11	0.01	0.36	0.45	−0.22	−0.17
Gln	0.06	−0.12	0.10	−0.22	−0.07	0.22	−0.27	0.06	0.54 ^a^	−0.43 ^a^	−0.12
His	0.15	−0.05	−0.09	−0.24	−0.15	0.23	−0.08	−0.05	0.30	−0.14	0.05
Gly	0.08	−0.06	0.08	−0.23	−0.18	0.24	−0.04	0.15	0.60	−0.37 ^a^	−0.10
Thr	−0.15	0.07	0.08	−0.10	−0.03	0.14	−0.20	−0.09	−0.43	−0.14	0.11
Arg	0.13	−0.12	−0.04	−0.02	0.22	−0.12	−0.31	−0.19	−0.17	−0.19	−0.26
Ala	−0.08	−0.01	0.00	−0.15	0.20	−0.16	−0.22	0.18	0.29	−0.30	−0.21
Tyr	−0.39 ^b^	0.26	0.19	0.20	−0.34 ^b^	0.31	0.27	−0.01	−0.41 ^b^	−0.16	−0.12
Cys	0.30	−0.04	−0.12	−0.49 ^a^	−0.20	0.33 ^b^	−0.17	0.12	0.75 ^a^	−0.54 ^a^	−0.36 ^b^
Val	0.16	−0.05	−0.04	−0.25	−0.37 ^b^	0.44 ^a^	0.07	0.03	−0.40	−0.30	−0.14
Met	−0.27	0.23	0.12	−0.04	−0.17	0.27	−0.11	0.10	0.09	−0.24	0.00
Trp	0.01	−0.16	0.14	0.01	0.08	−0.03	−0.16	0.15	0.03	0.01	0.14
Phe	−0.33 ^b^	0.22	0.13	−0.02	−0.14	0.19	−0.02	0.10	−0.07	−0.18	0.01
Ile	0.10	0.09	0.03	−0.21	−0.38 ^b^	0.47 ^a^	0.05	0.03	0.13	−0.38 ^b^	−0.25
Leu	0.04	−0.03	0.00	−0.17	−0.30	0.36 ^b^	0.06	0.09	0.27	−0.23	−0.08
Lys	−0.23	0.01	0.28	0.17	−0.13	0.05	0.23	−0.07	−0.23	0.11	0.04
FRAP	0.39 ^b^	−0.19	−0.19	−0.53 ^a^	−0.23	0.35 ^b^	−0.11	0.02		−0.46 ^a^	−0.36 ^b^
GSSG/GSH	−0.42 ^b^	0.03	0.19	0.50 ^a^	0.37 ^b^	−0.56 ^a^	0.13	−0.15	−0.46 ^a^		0.60 ^a^
Cortisol	−0.40	0.15	0.04	0.49 ^a^	0.32 ^b^	−0.32 ^b^	−0.18	0.00	−0.36 ^b^	0.60 ^a^	

^a^ Significant at <0.05 probability level (blue color).^b^ Significant at <0.01 probability level (red color).

## Appendix A

**Table A1:** Ingredients, major nutrients, and analyzed composition of the experimental diets.

Dietary Treatment	C	VE	OLE	VEOLE
Alpha Tocopherol (µg/g)				
	28.88	139.09	21.69	140.83
Fatty acids (g/100 g total fatty acids)				
C14:0	1.49	1.32	1.33	1.44
C16:0	19.74	20.23	20.10	20.17
C16:1n-9	0.25	0.27	0.26	0.24
C16:1n-7	1.22	1.35	1.32	1.34
C17:0	0.58	0.60	0.58	0.60
C17:1	0.19	0.21	0.19	0.21
C18:0	7.67	8.14	7.97	8.40
C18:1n-9	31.74	31.94	31.82	31.02
C18:1n-7	2.21	2.24	2.25	2.54
C18:2n-6	31.72	30.41	30.87	30.81
C18:3n-3	2.04	2.09	2.14	2.02
C18:4n-3	0.15	0.15	0.15	0.16
C20:0	0.37	0.36	0.37	0.36
C20:1n-9	0.63	0.68	0.66	0.66
∑SAT ^1^	29.84	30.65	30.34	30.98
∑MUFA ^2^	36.05	36.48	36.31	35.81
∑PUFA ^3^	33.91	32.65	33.15	33.00

^1^ SAT: Sum of total saturated fatty acids; ^2^ MUFA: Sum of total monounsaturated fatty acids; ^3^ PUFA: Sum of total polyunsaturated fatty acids. Ingredients P-31 (20%): Barley (40%), wheat (27.51%), corn (14.58%), soya 47 (11.81%), fat 3/5 (2.63%), calcium carbonate (1.50%), lysine 50 (0.59%), salt (0.50%), premix (0.30%), monocalcium phosphate, (0.24%), L-threonine (0.11%), phytase 500 dry (0.10%), DL-methionine (0.06%), and acid surfactant (0.05%). Ingredients P-50 (80%): Wheat (35%), corn (23.0%), barley (10.22%), biscuit (10%), sunflower 35 (8%), triticale (4.19%), fat 3/5 (2%), glycerol (1.50%), palm kernel (1.40%), rice (1.26%), soya 47 (1.09%), lysine 50 liq (0.66%), calcium carbonate (0.55%), salt (0.40%), premix (0.30%), sodium bicarbonate (0.19%), L-threonine (0.09%), copinat (essential oils, 0.05%), acid surfactant (0.05%), enzymes β-xilanase (0.04%), and phytase 5000 liquid (0.01%). Calculated major nutrients (%): Dry matter (10.5), crude protein (13.5), crude fat (4.9), linoleic acid (1.2), crude fiber (4.4), ash (4.0), lysine (0.8), net energy (mcal/kg, 2441.4), and metabolic energy (mcal/kg 3357.0).

## References

[1] Finkel T., Holbrook N.J. (2000). Oxidants, oxidative stress and the biology of ageing. Nature.

[2] Yin J., Wu M., Xiao H., Ren W., Duan J., Yang G., Li T.J., Yin Y.L. (2014). Development of an antioxidant system after early weaning in piglets. J. Anim. Sci..

[3] Varricchio E., Coccia E., Orso G., Vittoria Lombardi R., Pasquale I., Paolucci M. (2019). Influence of polyphenols from olive mill wastewater on the gastrointestinal tract, alveolar macrophages and blood leukocytes of pigs. Ital. J. Anim. Sci..

[4] Benavente-García O., Castillo J., Lorento J., Ortuno A., Del Rio J.A. (2000). Antioxidant activity of phenolics extracted from *Olea europaea* L. leaves. Food Chem..

[5] Harsini S.G., Habibiyan M., Moeini M.M., Abdolmohammadi A.R. (2012). Effects of dietary selenium, vitamin E, and their combination on growth, serum metabolites, and antioxidant defence system in skeletal muscle of broilers under heat stress. Biol. Trace Elem. Res..

[6] Sarica S., Toptas S. (2014). Effects of dietary oleuropein supplementation on growth performance, serum lipid concentrations and lipid oxidation of Japonese quails. J. Anim. Physiol. Anim. Nutr..

[7] Hassen I., Casbianca H., Hosni K. (2015). Biological activities of the natural antioxidant oleuropein: Exceeding the expecteation—A mini-review. J. Funct. Foods.

[8] Hadrich F., Garcia M., Maalej A., Modes M., Isoda H., Feve B., Sayadi S. (2016). Oleuropein activated AMPK and induced insulin sensitivity in C2C12 muscle cells. Life Sci..

[9] Anter J., Fernandez-Bedmar Z., Villatoro-Pulido M., Demyda-Feyras S., Moreno-Millan M., Alonso-Moraga A., Muñoz-Serrano A., Luque de Castro M.D. (2011). A pilot study on the DNA-protective of olive-leaf extracts. Mutat. Res..

[10] Drira R., Chen S., Sakamoto K. (2011). Oleuropein and hydroxytyrosol inhibit adipocyte differentiation in 3T3-L1 cells. Life Sci..

[11] Wu C.H., Chen S.C., Ou T.T., Chyau C.C., Chang Y.C., Wang C.J. (2013). Mulberry leaf polyphenol extracts reduced hepatic lipid accumulation involving regulation of adenosine monophosphate activated protein kinase and lipogenic enzymes. J. Funct. Foods.

[12] Tutino V., Orlando A., Russo F., Notarnicola M. (2016). Hydroxytyrosol inhibits cannabinoid CB1 receptor gene expression in 3T3 -L1 preadipocyte cell line. J. Cell. Physiol..

[13] Bee G., Biolley C., Guex G., Herzog W., Lonergan S.M., Huff-Lonergan E. (2006). Effects of available dietary carbohydrate and preslaughter treatment on glycolytic potential, protein degradation, and quality traits of pig muscles. J. Anim. Sci..

[14] Dasgupta T., Hebbel R.P., Kaul D.K. (2006). Protective effect of arginine on oxidative stress in transgenic sickle mouse models. Free Radic. Biol. Med..

[15] Bannai M., Kawai N., Ono K., Nakahara K., Murakami N. (2012). The effects of glycine on subjective daytime performance in partially sleep-restricted healthy volunteers. Front. Neurol..

[16] Fernstrom J.D. (2013). Large neutral amino acids: Dietary effects on brain neurochemistry and function. Amino Acids.

[17] Dagnino-Subiabre A. (2019). Stress and western diets increase vulnerability to neuropsychiatric disorders: A common mechanism. Nutr. Neurosci..

[18] Brigelius-Flohé R., Traber M.G. (1999). Vitamin E: Function and metabolism. FASEB J..

[19] Ebeid T.A., Zeweil H.S., Basyony M.M., Dosoky W.M., Badry H. (2013). Fortification of rabbit diets with vitamin E or selenium affects growth performance, lipid peroxidation, oxidative status and immune response in growing rabbits. Livest. Sci..

[20] Wang X., Wu H., Long Z., Sun Q., Liu J., Liu Y., Hai C. (2016). Differential effect of Se on insulin resistance: Regulation of adipogenesis and lipolysis. Mol. Cell. Biochem..

[21] Leskovec J., Rezar V., Nemec A., Salobir J., Levart A. (2019). Antioxidative effects of olive polyphenols compared to vitamin E in piglets fed a diet rich in n-3 PUFA. Animal.

[22] Benzie I.F.F., Strain J.J. (1999). Ferric reducing antioxidant power assay: Direct measure of total antioxidant activity of biological fluids and modified version for simultaneous measurement of total antioxidant power and ascorbic acid concentration. Method Enzymol..

[23] Rey A.I., Daza A., López-Carrasco C., López-Bote C.J. (2006). Quantitative study of the alpha- and gamma-tocopherols accumulation in muscle and backfat from Iberian pigs kept free-range as affected by time of free-range feeding or weight gain. Anim. Sci..

[24] Buege J.A., Aust S.D. (1978). Microsomal lipid peroxidation. Method Enzymol..

[25] Bruce S.T., Tavazzi I., Parisod V., Rezzi S., Kochhar S., Guy P.A. (2009). Investigation of human blood plasma sample preparation for performing metabolomics using ultrahigh performance liquid chromatography/mass spectrometry. Anal. Chem..

[26] Segura J., López-Bote C.J. (2014). A laboratory efficient method for intramuscular fat analysis. Food Chem..

[27] Garcés R., Mancha M. (1993). One-step lipid extraction and fatty acid methyl-esters preparation from fresh plant tissues. Anal. Biochem..

[28] Ruíz J., Antequera T., Andrés A.I., Petron M.J., Muriel E. (2004). Improvement of a solid phase extraction method for analysis of lipid fractions in muscle foods. Anal. Chim. Acta.

[29] Raclot T., Groscolas R. (1993). Differential mobilization of white adipose tissue fatty acids according to chain length, unsaturation, and positional isomerism. J. Lipid Res..

[30] Hamden K., Allouche N., Damak M., Elfeki A. (2009). Hypoglycemic and antioxidant effects of phenolic extracts and purified hydroxytirosol from olive mil waste in vitro and in rats. Chem. Biol. Interact..

[31] Abdel-Raheem S.M., Mahmoud G.B., Senosy W., El-Sherry T.M. (2019). Influence of vitamin E and selenium supplementation on the performance, reproductive indices and metabolic status of ossimi ewes. Slov. Vet. Res..

[32] Moazzami A.A., Andersson R., Kamal-Eldin A. (2011). Changes in the metabolic profile of rat liver after α-tocopherol deficiency as revealed by metabolomics analysis. NMR Biomed..

[33] Luo C., Wang X.Q., Yin J.W., Chen S.J., Liu L.G. (2019). Assotiation between plasma selenium and the risk of impaired glucose regulation. Chin. J. Prev. Med..

[34] Jacobs E.T., Lance P., Mandarino L.J., Ellis N.A., Chow H.H.S., Foote J., Martinez J.A., Hsu C.H.P., Batai K., Saboda K. (2019). Selenium supplementation and insulin resistance in a randomized, clinical trial. BMJ Open Diabetes Res. Care.

[35] Andreadou I., Ilpiodromitis E.K., Mikros E., Constantinou M., Agalias A., Magiatis P., Skaltsounis A.L., Kamber E., Tsantili-Kakoulidou A., Kremastinos D.T. (2006). The olive constituent oleuropein exhibits anti-ischemic, antioxidative, and hypolipidemic effects in anesthetized rabbits. J. Nutr..

[36] Saleh A.A., Ebeid T.A. (2019). Feeding sodium selenite and nano-selenium stimulates growth and oxidation resistance in broilers. S. Afr. J. Anim. Sci..

[37] Amazan D., Rey A.I., Fernández E., López-Bote C.J. (2012). Natural Vitamin E supplementation in drinking water prevents oxidative stress in weaned piglets. Livest. Sci..

[38] Pulido R., Bravo L., Saura-Calisto F. (2000). Antioxidant activity of dietary polyphenols as determined by a modified ferric reducing/ antioxidant power assay. J. Agric. Food Chem..

[39] Bars-Cortina D., Lopez de las Hazas M.C., Benavent-Vallés A., Motilva M.J. (2018). Impact of dietary supplementation with olive and thyme phenols on alpha-tocopherol concentration in the muscle and liver of adult Wistar rats. Food Funct..

[40] Meister A. (1988). Glutathiones metabolism and its selective modification. J. Biol. Chem..

[41] Senthilkumar R., Viswanathan P., Nalini N. (2003). Effect of glycine on oxidative stress in rats with alcohol induced liver injury. Pharmazie.

[42] Seminotti B., da Rosa M.S., Fernandes C.G., Amaral A.U., Braga L.M., Leipnitz G., de Souza D.O., Woontner M., Koeller D.M., Goodman S. (2012). Induction of oxidative stress in brain of glutaryl-CoA dehydrogenase deficient mice by acute lysine administration. Mol. Genet. Metab..

[43] Strauss K.A., Brumbaugh J., Duffy A., Wardley B., Robinson D., Hendrickson C., Tortorelly S., Moser A.B., Puffenberger E.G., Rider N.J. (2011). Safety, efficacy and physiological actions of a lysine-free, arginine-rich formula to treat glutaryl-CoA dehydrogenase deficiency: Focus on cerebral amino acid influx. Mol. Genet. Metab..

[44] González-Santiago M., Martín-Bautista E., Carrero J.J., Fonollá J., Baró L., Bartolomé M.V., Gil-Loyzaga P., López-Huertas E. (2006). One-month administration of hydroxytyrosol, a phenolic antioxidant present in olive oil, to hyperlipemic rabbits improves blood lipid profile, antioxidant status and reduces aterosclerosis development. Atherosclerosis.

[45] Bochicchio D., Comellini M., Lambertini P., Marchetto G., Della Casa G. (2015). Selective mobilization of fatty acids in adipose tissue of heavy pigs. Animal.

[46] Hermier D., Mariotti F. (2018). Importance of protein and amino acid metabolism in the prevention and management of the metabolic syndrome. Modulation by n-3 fatty acids. Cah. Nutr. Diet..

[47] Lee B., Shim I., Lee H., Hahm D.H. (2018). Oleuropein reduces anxiety-like responses by activating of serotonergic and neuropeptide Y (NPY)-ergic systems in a rat model of post-traumatic stress disorder. Anim. Cells Syst..

[48] Shakirullah, Queshi M.S., Akhtar S., Khan R.U. (2017). The effect of vitamin E and selenium on physiological, hormonal and antioxidant status of Damani and Balkhi sheep submitted to heat stress. Appl. Biol. Chem..

[49] Delarue J., Matzinger O., Binnert C., Schneiter P., Chiolero R., Tappy L. (2003). Fish oil prevents the adrenal activation elicited by mental stress in healthy men. Diabetes Metab..

[50] Chen C.T., Kitson A.P., Hopperton K.E., Domenichiello A.F., Trépanier M.O., Lin L.E., Ermini L., Post M., Thies F., Bazinet R.P. (2015). Plasma non-esterified docosahexaenoic acid is the major pool supplying the brain. Sci. Rep..

[51] Nemeth M., Millesi E., Wagner K., Wallner B. (2018). Saliva cortisol responses to altered plasma PUFA patterns in guinea pigs. Br. J. Nutr..

